# Community-led comparative genomic and phenotypic analysis of the aquaculture pathogen *Pseudomonas baetica* a390T sequenced by Ion semiconductor and Nanopore technologies

**DOI:** 10.1093/femsle/fny069

**Published:** 2018-03-22

**Authors:** Ainsley Beaton, Cédric Lood, Edward Cunningham-Oakes, Alison MacFadyen, Alex J Mullins, Walid El Bestawy, João Botelho, Sylvie Chevalier, Shannon Coleman, Chloe Dalzell, Stephen K Dolan, Alberto Faccenda, Maarten G K Ghequire, Steven Higgins, Alexander Kutschera, Jordan Murray, Martha Redway, Talal Salih, Ana C da Silva, Brian A Smith, Nathan Smits, Ryan Thomson, Stuart Woodcock, Martin Welch, Pierre Cornelis, Rob Lavigne, Vera van Noort, Nicholas P Tucker

**Affiliations:** 1 Strathclyde Institute of Pharmacy and Biomedical Science, University of Strathclyde, 161 Cathedral Street, Glasgow, G4 0RE, UK; 2 Centre of Microbial and Plant Genetics, KU Leuven, Kasteelpark Arenberg 20, bus 2460, Leuven B-3001, Belgium; 3 Laboratory of Gene Technology, KU Leuven, Kasteelpark Arenberg 20, bus 2460, Leuven B-3001, Belgium; 4 Cardiff School of Biosciences, Cardiff University, Sir Martin Evans Building, Park Place, Cardiff CF10 3AX, UK; 5 Royal (Dick) School of Veterinary Studies, University of Edinburgh, Easter Bush Campus, Midlothian EH25 9RG, Scotland, UK; 6 UCIBIO/REQUIMTE, Laboratório de Microbiologia, Faculdade de Farmácia, Universidade do Porto, Rua de Jorge Viterbo Ferreira no. 228 Porto 4050-313, Portugal; 7 Laboratoire Microbiologie Signaux et Microenvironnement (LMSM), Université de Rouen, 55, rue St Germain, Evreux 27000, France; 8 Lower Mall Research Station, University of British Columbia, 2259 Lower Mall, Vancouver, BC V6T 1Z4, Canada; 9 Department of Biochemistry, University of Cambridge, Hopkins Building, Tennis Court Road, Cambridge CB2 1QW, UK; 10 Department of Plant and Microbial Biology, University of Zürich, Zürich 8008, Switzerland; 11 Department of Phytopathology, Center of Life and Food Sciences, Technical University of Munich, Weihenstephan D-85354, Germany; 12 Centre for Biomolecular Sciences, School of Life Sciences, University of Nottingham, Nottingham NG7 2RD, UK; 13 School of Plant Sciences, The University of Arizona, P.O. Box 210036, Forbes Building, 303 Tucson, Arizona 85721-0036, USA; 14 Department of Biological Chemistry, John Innes Centre, Colney Lane, Norwich NR4 7UH, UK

**Keywords:** *Pseudomonas*, aquaculture, whole genome sequencing, Oxford Nanopore MinIon, Ion Torrent, *Pseudomonas baetica*

## Abstract

*Pseudomonas baetica* strain a390T is the type strain of this recently described species and here we present its high-contiguity draft genome. To celebrate the 16th International Conference on Pseudomonas, the genome of *P. baetica* strain a390T was sequenced using a unique combination of Ion Torrent semiconductor and Oxford Nanopore methods as part of a collaborative community-led project. The use of high-quality Ion Torrent sequences with long Nanopore reads gave rapid, high-contiguity and -quality, 16-contig genome sequence. Whole genome phylogenetic analysis places *P. baetica* within the *P. koreensis* clade of the *P. fluorescens* group. Comparison of the main genomic features of *P. baetica* with a variety of other *Pseudomonas* spp*.* suggests that it is a highly adaptable organism, typical of the genus. This strain was originally isolated from the liver of a diseased wedge sole fish, and genotypic and phenotypic analyses show that it is tolerant to osmotic stress and to oxytetracycline.

## Teaser

Here, we present the high-quality draft genome of *Pseudomonas baetica*, a bacterial pathogen that has been demonstrated to cause disease in fish cultivated in aquaculture facilities.

## INTRODUCTION

In September 2017, the biannual conference on the biology of *Pseudomonas* was held in Liverpool, UK. All aspects of *Pseudomonas* biology were reported at the meeting from clinical to environmental microbiology. The vast majority of the *Pseudomonas* literature focuses on just a few organisms, most notably *Pseudomonas aeruginosa*, *P. fluorescens*, *P. putida* and *P. syringae*, although the genus is far more diverse than this. In order to contribute to addressing this imbalance, the genome of *P. baetica*, a recently described member of the genus, was sequenced for the Pseudomonas 2017 genomics forum. The resulting genome sequence has been analysed by members of the Pseudomonas 2017 community from across the globe using Slack as a collaboration tool (Perkel 2016).

*Pseudomonas baetica* strain a390 is one of five Gram-negative organisms that were isolated from a fish disease outbreak in an aquaculture facility in Huelva, Spain, in 2006 (López *et al.*^2012^). The infected fish were wedge sole (*Dicologlossa cuneata*), a flat fish that is widely consumed in Andalucía and parts of France where there is increasing interest in sustainable aquaculture of this species. Aquaculture not only provides a sustainable source of protein, but it is also an economically important industry around the world. European commission data reveals that 1.25 million tonnes of food are produced by aquaculture in the EU every year and that over 85 000 people are directly employed in the industry. A disadvantage to aquaculture systems is that the unusually high population density of the farmed fish leads to an elevated risk of infectious disease outbreaks. For this reason, antibiotics such as oxytetracycline (OT) are routinely used as growth promoters in aquaculture fish food pellets (Leal *et al.*^2017^). Other members of the *Pseudomonadaceae* are well known to cause disease in aquaculture scenarios, most notably *P. anguilliseptica* is a pathogen of both farmed and wild eels (Joh *et al.*^2013^).

*Pseudomonas baetica* was formally described in 2012 using a combination of 16S rRNA sequencing and phenotypic analyses, allowing a biochemical profile to be generated (López *et al.*^2012^).

This analysis revealed that *P. baetica* is a member of the *P. fluorescens* group and is particularly closely related to *P. koreensis*. Although infected wedge sole showed no obvious visual signs of *P. baetica* infection, intraperitoneal injection and immersion infection assays yielded 100% and 10% mortality rates, respectively (López *et al.*^2012^). Subsequent infection assays with a variety of other fish species demonstrated that *P. baetica* caused higher mortality in wedge sole than other species including sea bass and sea bream, and that there is a temperature-dependent effect on virulence (López *et al.*^2017^). In order to rapidly identify *P. baetica* contamination in an aquaculture setting, López *et al.* (2017) have developed immunological and molecular detection assays.

Although *P. baetica* was originally described from an aquaculture infection scenario, a number of recent studies have identified this species and close relatives such as the newly described *P. helmanticensis* and *P. granadensis* in diverse environments in the rhizosphere including bean roots in Iran, and forest soils in Spain (Ramírez-Bahena *et al.*^2014^; Pascual *et al.*^2015^; Keshavarz-Tohid *et al.*^2017^). This suggests that *P. baetica* is more likely to be an opportunistic pathogen of fish as opposed to a genuine marine pathogen. We hypothesised that the draft genome sequence of *P. baetica* would reveal the genetic basis for the traits required to cause disease in wedge sole. The aim of this study is to provide genomic insights into the biology of *P. baetica* and to use this genome as a reflection on the diverse interests of the *Pseudomonas* community.

## MATERIALS AND METHODS

### Bacterial strains and genome sequence accessions

*Pseudomonas baetica* a390T originally isolated from the liver of wedge sole fish, *D. cuneate*, and described by Lopez *et al*. (2012), was obtained from DSMZ (DSM no. 26532) and is considered to be the type strain. Unless stated otherwise, all phenotypic comparisons were performed against *P. fluorescens* PF01, *P. putida* KT2440 and *P. aeruginosa* PA14. This Whole Genome Shotgun project has been deposited at DDBJ/ENA/GenBank under the accession PKLC00000000. The version described in this paper is version PKLC01000000. Raw sequencing reads were also deposited and can be found with accession numbers SRR6792524 and SRR6792523 for the Nanopore and Ion Torrent reads, respectively.

### DNA extraction, library preparation and Ion Torrent PGM Sequencing

DNA extraction was carried out using ISOLATE II genomic DNA kit (BIOLINE) using the standard protocol for bacterial cells. Fragmentation was carried out using the standard protocol from NEBNext Fast DNA Fragmentation & Library Prep Set for Ion Torrent and adapter-ligated DNA was prepared using barcode set 6. Size selection was carried out using the E-gel ibase (Invitrogen), and analysed using the 2100 Bioanalyzer (Agilent Genomics). A fragment size of 424 bp was carried forward. Template amplification and enrichment were carried out using the standard Ion Torrent protocol using the Ion OneTouch 2 system (Thermo-Fischer). The standard sequencing protocol for Ion Torrent was used and DNA library was loaded onto an Ion316 chip v2 with an ISP loading percentage of 87%.

### Nanopore sequencing

Long reads by Nanopore sequencing were obtained using a *MinION* sequencer from Oxford Nanopore Technology (ONT) with the goal to improve the genome assembly of *P. baetica.* The strain was grown in LB medium and grown at 30°C to an OD_600_ of 0.850 and its genomic DNA was isolated using the Mo Bio DNAeasy UltraClean Microbial kit. Quality of the gDNA was checked using a NanoDrop and an agarose gel for integrity. The gDNA was sheared mechanically to an average fragment size of 10 kbp using a Covaris *gTube.* The sequencing library was prepared using the 1D ligation protocol from ONT with native barcoding of the sample. The result was sequenced on a R9.4 flowcell for a period of 8 h.

### Combined Genome Assembly of Ion PGM and Nanopore Reads

The Ion PGM reads were checked for quality using *FastQC* and processed with *BBDuk* in order to remove potential adapter contamination, size exclusion (50> read length <250), and for trimming (phred score < 28) (Andrews 2014). Basecalling of the Nanopore reads was performed with the software *Albacore* v2.1.3 from ONT, and the barcodes were removed using *Porechop*. Read length distribution and quality of the Nanopore reads was assessed using *NanoPlot*.

The genome was first assembled with *SPAdes*, version 3.11.1 using default parameters (Bankevich *et al.*^2012^) using Ion PGM reads only, then with *Unicycler*, version v0.4.3 (Wick *et al.*^2017^) also using default parameters with both Ion PGM and Nanopore reads. For the latter, the genome was polished with Racon (Vaser *et al.*^2017^) and Pilon (Walker *et al.*^2014^). The quality of both assemblies was assessed with *QUAST* (Gurevich *et al.*^2013^).

### Genomic island prediction and genome visualisation

The Ion PGM and the hybrid assemblies were annotated with *Prokka* (Seemann 2014) using a custom protein database built from the *Pseudomonas* genus. The annotations were then searched for prophages using *PHASTER* (Arndt *et al.*^2016^), and genomic islands using the *IslandViewer* and antiSMASH (Bertelli *et al.*^2017^; Blin *et al.*^2017^). For the latter, the reference genome used for the alignment was that of *P. koreensis* strain D26 as it was deemed phylogenetically close to *P. baetica.* CCT maps were created using CCT version 1.0 using default settings with the exception of the following commands; -t –cct –custom scale_blast=F _cct_blast_thickness=60 backboneThickness=10 tickThickness=15 rulerFontSize=100 featureThickness=150 tickLength=30 legend=F details=F. A features file was used for the ‘beige blocks’, and a ‘labels_to_show’ file was used to label the CCT map (Grant, Arantes and Stothard 2012).

### Phylogenetic analysis

Trees were assembled using Mash v1.1.1 (Ondov *et al.*^2016^) fast genome and metagenome distance estimation using the MinHash algorithm, and visualised in FigTree v1.4.3 (Rambaut 2012, also available via GitHub). Reference genomes were obtained from NCBI. Average nucleotide identity (ANI) was performed using PyANI (Gupta *et al.*^2016^). Data were processed using CLIMB (Connor *et al.*^2016^).

### Azocasein assay

Overnight cultures of *P. aeruginosa* UCBPP-PA14, *P. baetica* a390T, *P. fluorescens* Pf0-1 and *P. putida* KT2440 were centrifuged at 12 000× *g* for 5 min and 100 μl of supernatant was pipetted into centrifuge tubes containing 400 μl of reaction mixture; 200 μl of 2% azocasein in 0.5% bicarbonate sodium buffer and 200 μl of 0.5% sodium bicarbonate buffer. The reaction mixture was then incubated at 30°C for 10, 70 and 140 min.

Following incubation, 0.5 ml of 10% trichloroacetic acid was added to terminate the reaction. Tubes were then vortexed for 5 min followed by centrifuging for 5 min at 4°C and 10 000× *g.* Five hundred microlitre of supernatant was then added to cuvettes containing 500 μl of 0.5 M NaOH, and absorbance was measured at 440 nm in an Ultrospec 3100pro spectrophotometer. All samples were run in triplicate.

### Skim milk assay

Five microlitre of overnight cultures of *P. aeruginosa* UCBPP-PA14, *P. baetica* a390T, *P. fluorescens* Pf0-1 and *P. putida* KT2440 were inoculated on to MOPS minimal media agar plates, supplemented with dehydrated skimmed milk and incubated for 72 h at 30°C. Photographs were taken at 24-h time intervals. Iron supplement, FeCl_2_, was not included in the 10× stock, and 5g/L of agar and skim milk were added in order to observe clear halo formation.

### OT resistance assay

*Pseudomonas baetica* was tested for antibiotic sensitivity by agar dilution according to the European Committee for Antimicrobial Susceptibility Testing guidelines (European Committee for Antimicrobial Susceptibility Testing (EUCAST) of the European Society of Clinical Microbiology and Infectious Diseases (ESCMID) 2000). Resistance to OT was tested due to its frequent use as a growth promoter in aquaculture (Leal *et al.*^2017^). Mueller-Hinton agar plates with final concentrations ranging from 0.125 to 128 μg mL^−1^ were inoculated with *P. baetica* a390T suspensions diluted in 0.85% NaCl to a turbidity equivalent of a 0.5 McFarland standard. Plates were incubated overnight at 30ºC. *Pseudomonas aeruginosa* PAO1 was used as a positive control.

## RESULTS

### Sequencing results and assembly

The initial Ion Torrent sequencing produced over 3.5 million barcoded reads with a mean read length of 299 bp. SPAdes assembly resulted in final contig number of 338. The Nanopore sequencing run yielded around 22 000 reads, of which 7771 passed quality control during the basecalling process. The average phred quality score of the reads is 11 and their length distribution was around 9 kbp (see Figure S1, Supporting Information). With a genome size of about 6.6 Mbp, estimated from an initial Ion PGM assembly, we infer a genome coverage of about 9× for the Nanopore sequencing data (60 Mbp), and 80× with Ion PGM data (540 Mbp after QC).

The hybrid approach that combines both the dataset of short Ion PGM reads with the long Nanopore reads significantly improved the genome assembly over that using Ion PGM reads only giving 16 contigs over 1000 bp (Table 1). Quast analysis of the combined assembly gives an N50 value of 973739 which is higher than 96% of all draft genomes in The Pseudomonas Genome database.

**Table 1.** Assessment of genome assembly quality for both approaches reveals a vast improvement in the assembly despite a low nanopore sequencing coverage (9X).

Metric	Ion PGM only	Hybrid
Assembly length	6602 908	6773 804
No. of contigs (>1000 bp)	338	16
N50	36 090	973 739
N75	19 947	745 006
Longest contig	122 046	1654 292

The Ion PGM-only and the hybrid assembly graphs were also inspected visually using *Bandage* (Wick *et al.*^2015^) in order to assess the fragmentation of the genome assemblies and the genomic organisation, including the presence of potential plasmids (see Figures S2 and S3, Supporting Information). After gene annotation with *Prokka*, plasmid-associated genes like recombinases, transposases and integrases were searched for and were not present or were not indicative of being plasmid associated. The high continuity assembly provided by the combinatorial approach of Ion Torrent and Nanopore technologies allowed for a comprehensive analysis of the genome.

### Phylogenetic placement and ANI of *P. baetica* a390T

**Figure 1.**
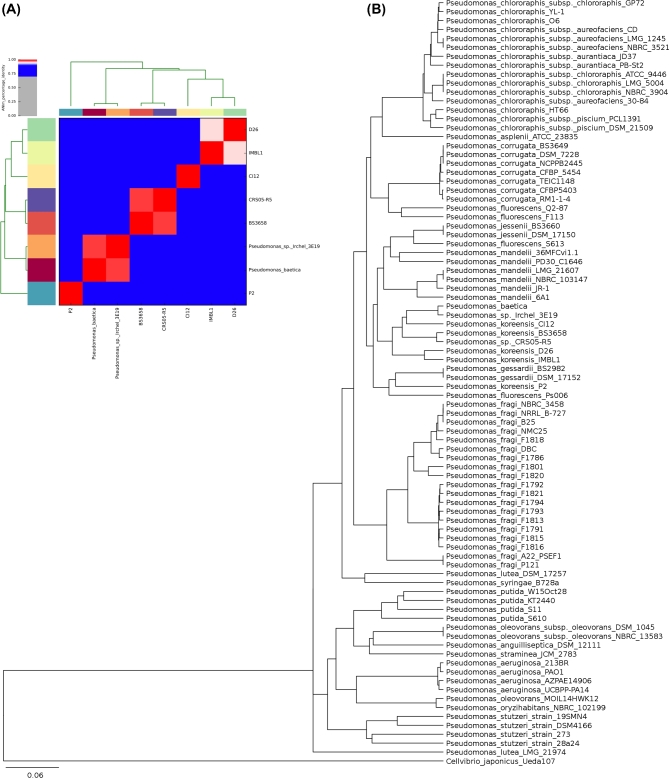
Placement of novel *baetica* genome in the genus *Pseudomonas* based upon ANI, and shared k-mers A) ANI heatmap generated the Python3 module pyani. The sequenced baetica genome, Pseudomonas_sp._Irchel_3E19 and reference genomes from the closely related *koreensis* subclade were subject to ANI analysis. B) A Mash-based tree generated from reference genomes (= 86) from 19 species clades, comprising the entire genus *Pseudomonas*. This tree was generated based upon the Jaccard index, calculated from shared k-mers. *Cellvibrio japonicum* Ueda107 was used as outgroup.

We have produced a phylogeny to place the sequenced *P. baetica* a390T genome against reference genomes from 19 species clades, comprising the entire genus *Pseudomonas* (Fig. 1B) (Gomila *et al.*^2015^). This was done using Mash, a pipeline that converts a genome into a sketch (or groups of k-mers). Sketches are then compared to produce a Jaccard index, which is generated based upon shared k-mers between genomes (Ondov *et al.*^2016^). Our phylogeny places *P. baetica* amongst the *P. koreensis* subclade, as previously shown in phylogenies constructed using partial 23S rRNA *P. koreensis* gene sequences. A *P. koreensis* phylogeny constructed in the same manner places *P. baetica* closest to *Pseudomonas sp.* Irchel 3E19, a strain isolated from a pond in Zurich, Switzerland, suggesting that this isolate is in fact a strain of *P. baetica* (Butaitė *et al.*^2017^). The next most closely related organism is *P. koreensis strain* CI12, which was recently co-isolated with *Bacillus cereus* from a soybean plant rhizosphere (Bravo, Lozano and Handelsman 2017). ANI was used to compare the whole genome sequence of *P. baetica* against the six available reference genomes for *P. koreensis* as well as strain Irchel 3E19, and confirms that it is most closely related to Irchel 3E19 and CI12, with an ANI of 98.02% and 89.6% similarity, respectively (Fig. 1A) (Arahal 2014). We also used JSpeciesWS as an independent ANI method and this analysis agreed with those described above (see supplementary ANI tables) (Richter *et al.*^2016^). Finally, BLAST was used to compare the *gyrB* and *rpoD* genes sequences of *P. baetica* a390T and CI12 against available partial *rpoD* and *gyrB* sequences available for *P. baetica* (López *et al.*^2012^). This showed a 93.6% similarity in *rpoD* and 95.0% similarity in *gyrB* between CI12 and the whole genome-sequenced *P. baetica* a390T.

### Genomic islands, biosynthetic gene clusters and prophages detected in the *P. baetica* genome

**Figure 2.**
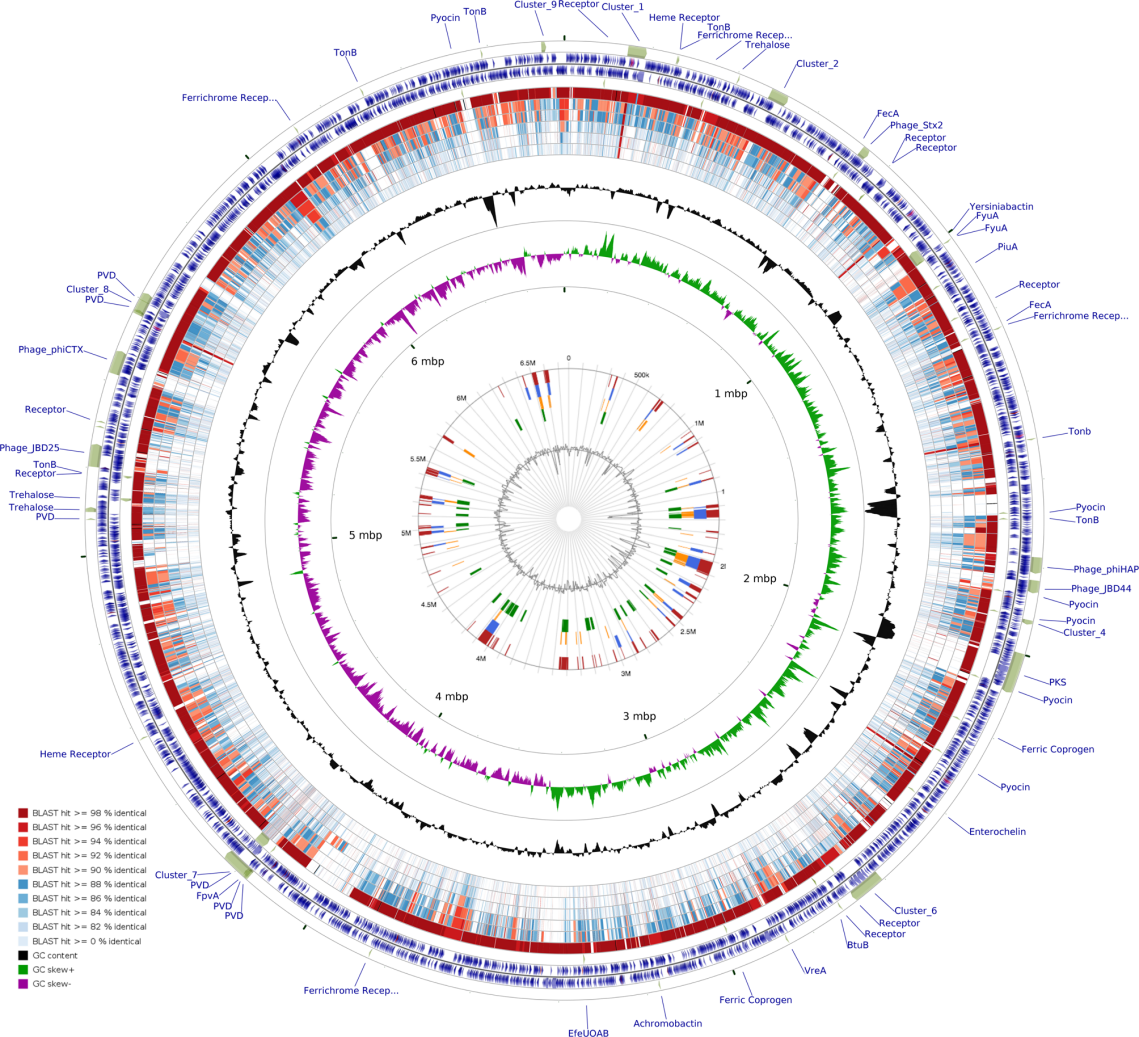
Overview of the genomic architecture of *P. baetica* a390T. CGView Comparison Tool was used to plot the percentage sequence identity of closely related strains. The outer labels and beige blocks refer to loci that are mentioned throughout the paper. The *P. baetica* a390T genome is split into the forward and reverse strands and the coding sequences are represented by blue arrows. The next 6 rings represent the percentage BLAST identity of the genomes of the following closely related strains; from the outside ring to inside the order is *P. sp Irchel 3E19*, *P. koreensis CI12*, *P. koreensis D26*, *P. fluorescens SBW25*, *P. putida KT2440*, *P. aeruginosa UCBPP-PA14*. The percentage identity is indicated by the colour of the 6 BLAST rings as indicated by the key on the bottom left hand side of the figure. Moving inwards, the next two rings indicate the changes in %GC content and the GC skew respectively. Finally, the central diagram indicates the genomic islands predicted by the IslandViewer package where Red = predicted by multiple methods, blue = IslandPath-DIMOB, orange = SIGI-HMM and green = IslandPick.

Genomic islands can be readily detected when long contiguous regions are assembled. As such, searching for prophages in the highly fragmented, ion PGM-only assembly did not yield any results, whereas inspection of the hybrid assembly showed four intact prophages in the genome of *P. baetica*, as well as five incomplete ones (Table 2a). Two integrative conjugative elements were also detected. A combination of IslandViewer and antiSMASH were also used to predict genomic islands and the secondary metabolite biosynthetic potential of *P. baetica* (Bertelli *et al.*^2017^; Blin *et al.*^2017^)*.* These results are summarised on the CG View Comparison Tool map in Fig. 2, and a more detailed summary of the antiSMASH results is provided in Table 2b (Grant, Arantes and Stothard 2012). Many of the loci referred to throughout this manuscript are labelled around the CCT map in Fig. 2 as well as the results of IslandViewer. The CCT BLAST analysis confirms the close phylogenetic relationship between *P. baetica* a390 and *Irchel* 3E19 (Fig. 2). Of the nine biosynthetic gene clusters (BGCs) predicted by antiSMASH, four are Nrps (non-ribosomal peptide synthetases) biosynthetic gene clusters. Clusters 3 and 8 had relatively low similarities to the nearest known clusters; however, cluster 6 shared a high similarity with an orfamide BGC with 80% of genes in this cluster sharing homology with the putisolvin biosurfactant BGC. Putisolvin has been implicated in motility (Cárcamo-Oyarce *et al.*^2015^) and our preliminary experiments have demonstrated that *P. baetica* is capable of both swimming and swarming (see Figure S4, Supporting Information). Since putisolvin biosynthesis in certain strains of *P. putida* is known to be a quorum sensing (QS)-regulated phenotype, we searched for homologues of known QS systems in the *P. baetica* genome (Dubern *et al.*^2008^). No convincing homologues of the LasR or RhlR regulated systems from *P. aeruginosa*, or the PpuI, PpuR, PpuA, RsaL system from *P. putida* were found.

**Table 2a.** List of prophage regions detected in the hybrid assembly.

Contig no.	Length	Completeness	Score	No. of proteins	Region position	Most common phage	GC %
1	6.7 Kbp	Questionable	70	6	681 684–688 458	PHAGE_Stx2_c_1717_NC_011357(3)	53.77%
2	6.5 Kbp	Incomplete	40	10	512 098–518 615	PHAGE_Entero_phi92_NC_023693(2)	60.11%
2	10.2 Kbp	Incomplete	30	8	742 363–752 628	PHAGE_Clostr_phiCT453A_NC_028991(3)	56.63%
2	5.5 Kbp	Incomplete	60	6	925 750–931 322	PHAGE_Stx2_c_1717_NC_011357(3)	54.15%
3	40.7 Kbp	Intact	150	48	383 269–424 017	PHAGE_Vibrio_VP882_NC_009016(12)	58.74%
3	57.2 Kbp	Intact	150	49	430 072–487 323	PHAGE_Vibrio_vB_VpaM_MAR_NC_019722(8)	58.69%
4	35.1 Kbp	Intact	150	42	357 957–393 153	PHAGE_Pseudo_phiCTX_NC_003278(18)	57.32%
4	50.1 Kbp	Intact	150	70	564 492–614 686	PHAGE_Entero_Arya_NC_031048(10)	56.98%
5	21.3 Kbp	Incomplete	40	11	26 364–47 679	PHAGE_Pseudo_vB_PsyM_KIL1_NC_030934(6)	49.79%
5	8.7 Kbp	Incomplete	50	13	540 790–549 505	PHAGE_Bacill_BMBtpLA_NC_028748(1)	57.37%

**Table 2b.** Secondary metabolite biosynthetic clusters predicted by antiSMASH.

Cluster	Type	Cluster size (bp)	Most similar known biosynthetic gene cluster	Percentage similarity
1	Unknown	43 395	Mangotoxin	71
2	Arylpolyene	43 613	APE_Vf	40
3	Nrps	63 804	Nataxazole	11
4	Bacteriocin/Ripp	10 887	–	–
5	Transatpks-Nrps	94 211	Sorangicin	13
6	Nrps	77 485	Orfamide/Putisolvin	70
7	Terpene-Nrps	73 119	Pyoverdine	16
8	Nrps	52 998	Pyoverdine	22
9	Bacteriocin/Ripp	10 845	–	–

### Bacteriocins, tailocins and modular bacteriocins of *P. baetica*

Pseudomonads are capable of producing a variety of antagonism-mediating peptides and proteins. A subset of these molecules are bacteriocins, and assist in the elimination of phylogenetically related competitors (Ghequire and de Mot 2014). Historically, these compounds have been mainly studied in *P. aeruginosa*, there termed pyocins (Michel-Briand and Baysse 2002), but they have been equally detected and characterized in a number of other *Pseudomonas* species. Evolutionarily related to bacteriophage tails, tailocins are functional standalone units—rather than defective prophages—lacking an accompanying phage head (Ghequire and de Mot 2015). *Pseudomonas* phage tail-like bacteriocins are typically recruited to *trpE-trpG* and *mutS-cinA* intergenic regions (Ghequire and de Mot 2015). Inspection of the corresponding regions in *P. baetica* reveals the presence of two adjacent R-type gene clusters at the *mutS-cinA* locus. Phylogenetic assessment and gene synteny is indicative of an Rp3-Rp4 organisation, and a similar tailocin configuration was recently described in *P. chlororaphis* 30-84 (Dorosky *et al.*^2017^). An interesting observation is that four putative tail fibres are likely present in the Rp4 cassette of *P. baetica*, whereas usually only one or two of these can be detected in tailocins.

Referred to as S-type pyocins in *P. aeruginosa*, modular bacteriocins represent a heterogeneous group of polymorphic toxins (Zhang *et al.*^2012^; Jamet and Nassif 2015), comprising a receptor-binding domain, a segment enabling membrane transfer and a toxin domain at the carboxy-terminus (Ghequire and de Mot 2014). To protect from self-inhibition, bacteriocin producer strains co-express an immunity gene, typically encoded adjacent to the bacteriocin killer gene. *Pseudomonas* nuclease bacteriocins (DNase, tRNase, rRNase) are consistently equipped with a central pyocin_S domain (Pfam06958), and suggested to play a pivotal role in translocation of the toxin to the cytoplasm (Ghequire and de Mot 2014; Sharp *et al.*^2017^). Searching for this domain revealed an array of putative modular bacteriocins in *P. baetica*, seven of which host an HNH-DNase and three an rRNase domain. In one bacteriocin, a putative (non-HNH-type) DNase homologous to the killer domain found in carocin D—but distinct from the non-HNH-type in pyocin S3—was identified.

Two other predicted *P. baetica* bacteriocins with homology to a toxin module of a contact-dependent inhibition cassette (type IV) in *Burkholderia pseudomallei* were also detected. No bacteriocins acting at the periplasmic level (colicin M-like, Pfam14859; pesticins, Pfam16754; or pore formers (ColIa/ColN), Pfam01024) could be retrieved.

### Lipid A and core oligosaccharide biosynthesis gene clusters

Putative homologues of the confirmed lipid A biosynthesis (Raetz pathway) enzymes in *P. aeruginosa* PAO1 could be identified in the sequenced genome. Comparison to different *Pseudomonas* proteomes revealed sequence identities of the putative proteins ranging from 59% to 97%. Those high-sequence identities and the hydrocarbon ruler motive in putative LpxA (UDP-N-acetylglucosamine acyltransferase) hint towards a lipid A with a C10 acyl chain at position 3 and 3^΄^ (Smith *et al.*^2015^). Besides, C12 secondary acyl chains or C12 primary acyl chains at position 2 and 2^΄^ are possible due to sequence homology of putative LpxD and the presents of possible HtrB1 and HtrB2 homologs (Lam *et al.*^2011^; Hittle *et al.*^2015^). In general, it is likely that *P. baetica* possesses a lipid A structure similar to known structures from *P. aeruginosa* when possible post-synthesis modifications are not taken into account (Knirel *et al.*^2006^).

Further genome analysis resulted in the identification of a gene cluster homologous to the core oligosaccharide gene cluster which was described and studied in *P. aeruginosa*. Direct comparison of the cluster revealed that *P. baetica* lacks homologues of the *P. aeruginosa* genes PA4998 (putative kinase) and PA5008 (heptose kinase), which possibly suggests a less phosphorylated core oligosaccharide (Lam *et al.*^2011^). Reciprocal BLAST searching of different *Pseudomonas* proteomes resulted in sequence identities between the wide range of 14% and 98%. The highest overall sequence similarity was observed to *P. fluorescens* Pf0-1 core oligosaccharide biosynthesis proteins, ranging from 85% to 98% which is consistent with the phylogeny. In general, the cluster structure and presence of putative glycosyltransferases (WapR, WapH, WaaG, WaaC, WaaF) indicates the presence of an inner core oligosaccharide structure similar to *P. aeruginosa* (Knirel *et al.*^2006^).

### Extracellular protease and effector secretion by *P. baetica*

Extracellular protease secretion is an important virulence determinant of *P. aeruginosa*. In order to determine if *P. baetica* has a similar phenotype, azocasein and skimmed milk protease assays were performed with *P. aeruginosa* UCBPP-PA14*, P. baetica, P. fluorescens* Pf01, and *P. putida* KT2440*.* As expected, *P. aeruginosa* UCBPP-PA14 secreted prodigious amounts of protease (see Figures 5 and 6, Supporting Information). *Pseudomonas baetica* a390T secreted significantly less protease than *P. aeruginosa* but marginally more than the other strains. This phenotype was most evident on MOPS minimal milk agar after 72 h (see Figure S6b, Supporting Information).

A major virulence determinant in Gram-negative pathogens is the ability to inject effector proteins into host cells using a type III secretion system (T3SS) (Hauser 2009). BLAST analysis using the *P. aeruginosa* Psc T3SS proteins revealed a T3SS to be present in the *P. baetica* genome, providing a possible explanation for the virulence of this strain as hypothesised above.

### OT resistance in *P. baetica*

Following overnight incubation, antibiotic plates were examined for bacterial growth, and the minimum inhibitory concentration (MIC) of OT for *P. aeruginosa* PAO1 was determined as 1 μg mL^−1^, a quarter that of *P. baetica* a390T*.* OT is a protein synthesis-inhibiting antibiotic belonging to the tetracycline class, and is one of the most commonly used antibiotics in aquaculture (Leal *et al.*^2017^).

When *P. baetica* was cultured on media containing OT, it was able to grow in the presence of higher concentrations than *P. aeruginosa* PAO1. The minimum inhibitory concentration of OT required to inhibit the growth of *P. baetica* was determined as 4 μg mL^−1^. The ability of *P. baetica* to tolerate higher concentrations of OT than a lab strain of *Pseudomonas* may be due to the use of the antibiotic in aquaculture, especially as an additive in fish food. OT resistance (OT^R^) has been observed previously in bacterial species colonising pelletised fish food in Chile, and also in half of commercial fish food samples analysed in a 1995 study of OT^R^ in fish farm sediments (Kerry *et al.*^1995^; Miranda *et al.*^2003^).

### Glycerol-3-phosphate utilisation in *P. baetica*

A notable characteristic which differentiates *P. baetica* from closely related *Pseudomonas* species is an inability to grow on glycerol-3-phosphate (G3P) as a sole carbon source (López *et al.*^2012^). Several other pseudomonads, such as *P. fluorescens* and *P. aeruginosa* can use G3P as sole carbon sources. However, *P. baetica* can use glycerol as a sole carbon source. This suggested that *P. baetica* suffers from a specific deficit in G3P uptake, not metabolism. Uptake of G3P is known to occur using the specific MFS transporter GlpT (Fann, Busch and Maloney 2003).

The newly sequenced genome allowed us to examine the *P. baetica* growth deficiency on G3P in greater detail. Remarkably, *P. baetica* appears to harbour a *glpT* homologue, which is quite similar to GlpT from other pseudomonads (see Figure S7, Supporting Information). The structure of GlpT has been elucidated for *Escherichia coli*, allowing us to understand which residues are important for substrate recognition (Huang *et al.*^2003^). All residues which have been identified as important for functionality in previous studies are unchanged in *P. baetica*. Mutation of K80 to alanine killed heterologous G3P-Pi transport of the protein reconstituted into proteoliposomes, and mutation of H165 resulted in a transport rate that was only ∼6% of that catalysed by wild-type protein (Law *et al.*^2008^). These data suggest that the presence of a *glpT* homolog does not necessarily equate to G3P uptake, highlighting the necessity of experimental validation. This result may be due to a lack of induction of the *P. baetica glpT* gene upon exposure to G3P.

The broad-spectrum bactericidal antibiotic, fosfomycin, is taken up actively into bacterial cells via GlpT and the glucose-6-phosphate transporter UhpT, and inhibits the initial step in cell wall synthesis (Kahan *et al.*^1974^). Inactivation of *glpT* was shown to confer increased fosfomycin resistance in *P. aeruginosa*, with an apparent lack of fitness cost. These authors isolated 10 independent spontaneous mutants, obtained on LB-fosfomycin, which harboured mutations in the *glpT* gene. These mutants included several deletions, frameshifts and three single amino acid changes, Gly_137_ to Asp, Thr_336_ to Pro and Met_366_ to Ile (Castañeda-García *et al.*^2009^). Indeed, mutations in the *Staphylococcus aureus glpT* gene were recently shown to be the major determinant of fosfomycin resistance in this organism (Xu *et al.*^2017^). The high fosfomycin resistance and low biological fitness cost resulting from the loss of G3P uptake suggests that *P. baetica* may have an evolutionary advantage in a fosfomycin-rich environment. Future work will be necessary to confirm precisely how G3P uptake has been lost in *P. baetica*.

### Orthologs of MexAB-OprM and MexEF-OprN resistance-nodulation cell division efflux systems in *P. baetica*

The MexAB-OprM and MexEF-OprN systems are members of the resistance-nodulation cell division multidrug efflux pump family (Poole 2011). BLASTp was used to compare the amino acid sequences encoded by the *mexEF-oprN* and *mexAB-oprM* operons in *P. aeruginosa* strain PAO1 with homologues in *P. baetica*.

MexAB-OprM is known to play a key role in efflux-mediated resistance to a wide variety of compounds such as antibiotics and solvents in *P. aeruginosa* (Li, Zhang and Poole 1998; Poole 2011). Similarly, the MexEF-OprN efflux substrates include antibiotics and the QS precursor 4-hydroxy-2-heptylquinoline (Lamarche and Déziel 2011; Llanes *et al.*^2011^). *Pseudomonas baetica* sequences producing significant alignment to MexAB-OprM and MexEF-OprN were identified with between 69%–79% and 72%–88% identity to *P. aeruginosa* strain PAO1, respectively. Interestingly, the arrangement of the *mexAB-oprM* operon in *P. baetica* bears more similarity to *P. fluorescens*, where the adjacent regulatory gene encodes a member of the TetR regulator family, unlike *P. aeruginosa* which encodes the MarR family regulator MexR in this position. However, it should be noted that *P. baetica* does have a MexR homologue located adjacent to a third efflux operon which has significantly less homology (8%–31%) to *mexAB-oprM*.

### Outer membrane porins in *P. baetica*

In addition to the Mex-associated porins, the *P. baetica* genome was searched for genes coding for other outer membrane porins. There are 26 porin genes in *P. aeruginosa* involved in different important biological functions: outer membrane stability (OprF, OprH) and nutrient uptake (OprB, OprP, and the porins of the OprD family), which have recently been reviewed (Chevalier *et al.*^2017^). The *P. baetica* genome revealed only 17 porin genes, but the major porins described in *P. aeruginosa* are conserved. The *oprF* gene coding for the major *Pseudomonas* porin is present in *P. baetica* and the genomic context as well: *estX*-*menG*-*cmaX*-*cfrX*-*cmpX*-*sigX*-*oprF* where EstX is an esterase, MenG a RNAse inhibitor, CmaX a CorA-like magnesium transporter, CfrX a putative anti-sigma factor, CmpX a mechano-sensitive channel, and SigX an ECH-sigma factor. OprF has a *C*-terminal periplasmic domain interacting with peptidoglycan and a barrel *N*-terminal domain and it is a major contributor to the envelope stability. Other conserved porin genes are *oprG* and *oprH*, which code for small porins with only eight strands. OprG belongs to the OmpW family and has been proposed to be involved in Fe^2+^ diffusion to the periplasm (Catel-Ferreira *et al.*^2016^). OprH is induced by low Mg^2+^ and contributes to the outer membrane stability via its interaction with LPS (Kucharska *et al.*^2015^). As in *P. aeruginosa*, *P. baetica* also has three genes for the glucose porin OprB, including one in the conserved *gltBFGKoprB* operon (Chevalier *et al.*^2017^). Surprisingly, the *P. baetica* genome does not contain genes for the OprO and OprP phosphate uptake porins of *P. aeruginosa*. The OprD family comprises 19 members in *P. aeruginosa* sub-divided into two sub-families, the OccD being involved in the uptake of basic amino acids and the OccK for the uptake of negatively charged cyclic molecules (Eren *et al.*^2012^). The comparison with *P. aeruginosa* porins via BLASTP is complicated by the fact that many members of the OprD family show significant relatedness. Nevertheless, the survey of the *P. baetica* genome revealed five members of the OccD and six of the OccK family.

### Osmotic stress resistance of *P. baetica*

Since *P. baetica* a390T was originally isolated from a marine aquaculture scenario, we have investigated its genotypic and phenotypic ability to overcome salt stress. Trehalose is a stress-relieving disaccharide which accumulates within the bacterial cell in response to stress. It has been widely studied for its role as a compatible solute that confers protection against osmotic stress. It is thought that trehalose rehydrates the cell following water loss, preventing the loss of cellular functions (De Smet *et al.*^2000^). It has also been reported that trehalose is important for pathogenicity of *Pseudomonas* spp*.* (Freeman, Chen and Beattie 2010; Djonović *et al.*^2013^). Bacteria possess three main pathways for trehalose biosynthesis: OtsAB, TreYZ and TreS.

The OtsAB pathway is the most common route of trehalose biosynthesis in bacteria. OtsA catalyses the synthesis of trehalose 6-phosphate from UDP-glucose or ADP-glucose and glucose 6-phosphate. The phosphate group is then removed by OtsB to give trehalose (De Smet *et al.*^2000^). The TreYZ and TreS pathways produce trehalose using different substrates. TreY converts reducing maltosyl units of maltooligosaccharides to give α-1,1 terminal moieties which are then cleaved by TreZ to produce trehalose. The TreS pathway isomerises maltose into trehalose, catalysed by the trehalose synthase TreS, but its utility in vivo is context-dependent (Miah *et al.*^2013^).

Comparative genomics have shown that in common with other *Pseudomonas* spp., *P*. *baetica* lacks the genes for the OtsA/OtsB pathway. However, *P. baetica* possesses the enzymes of the TreYZ and TreS pathways.

Although TreS is known to be capable of generating trehalose in some bacteria, it is predicted that the function of TreS in *P. baetica* is to catabolise trehalose. Phenotypic observations suggest that the ability of *P. baetica* to utilise trehalose as a sole carbon source is dependent on other conditions. In a study by López *et al.*, it was reported that *P. baetica* was unable to utilise trehalose. Because of its importance to osmotic stress, we repeated the Omnilog analysis for trehalose and observed an intermediate phenotype and further analysis on defined media shows that *P. baetica* is capable of utilising trehalose.

The *treS* gene is fused with the maltokinase-encoding *pep2* gene (Chandra, Chater and Bornemann 2011), consistent with a TreS/Pep2 fusion protein converting trehalose into α-maltose 1-phosphate (M1P) (Miah *et al.*^2013^). M1P is expected to be acted upon by GlgE and GlgB to produce a glycogen-like α-glucan, given that the *treS*/*pep2*, *glgE* and *glgB* genes are clustered.

Other *Pseudomonas* spp. such as *P. fluorescens* and *P. syringae* possess an additional homologue of an unfused *treS* gene emphasising the importance of trehalose production in these organisms. Interestingly, this additional homologue is absent in *P. baetica*. *P. fluorescens* and *P. syringae* likely benefit from being able to control the synthesis of trehalose under more varied conditions.

We hypothesised that *P. baetica* was capable of causing disease in marine fish because it had greater salt tolerance than other pseudomonads; however, this proved to be incorrect. Our data show that all of the pseudomonads tested here are capable of growth in NaCl concentrations relevant to the marine environment (see Figures S8 and S9, Supporting Information).

**Figure 3.**
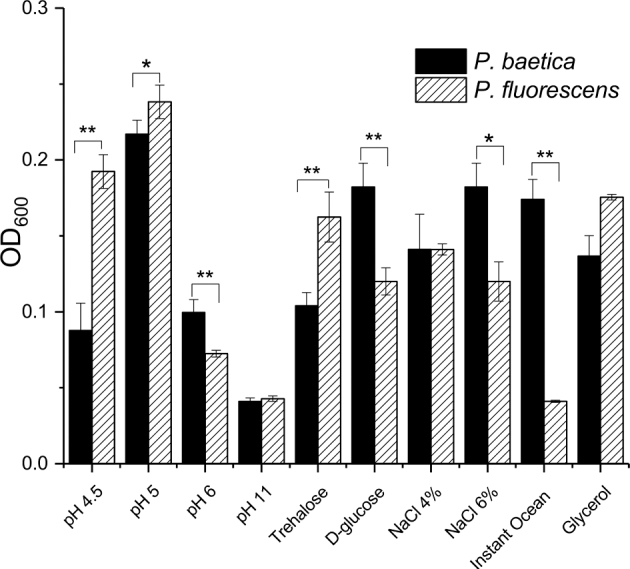
Stress tolerance and growth characteristics of *P. baetica* a390T. Graph of end point growth measured as OD_600_ after 24 h incubation at 30°C in *Pseudomonas* minimal media (L-glutamine 5%, K_2_HPO_4_ 1.5%, MgSO_4_ 0.2%, 20mM glycerol or other carbon source as indicated) with pH or salt concentration adjusted as indicated. Data represented mean ± SD. *P < 0.05, **P < 0.01 (determined by two-way *t*-test).

In addition to salt tolerance experiments, *P. baetica* and *P. fluorescens* were also tested for their ability to grow in minimal media with the addition of Instant Ocean which has similar levels of most trace elements, nutrients and alkalinity to sea water samples (Fig. 3). *Pseudomonas baetica* grows well in Instant Ocean while *P. fluorescens* has minimal growth suggesting that *P. baetica* may have some adaptation to the marine environment. Analysis of both genomes using SEED viewer showed that *P. baetica* has a homologue of the *OsmY* gene which has a protein modification function during osmotic stress whereas *P. fluorescens* does not (Métris, George and Ropers 2017).

### Iron uptake genes in *P. baetica*

The *P. baetica* genome was searched for genes coding for siderophore biosynthesis and uptake proteins as well as those involved in Fe^2+^ uptake. As expected, genes for the biosynthesis and uptake of the fluorescent siderophore pyoverdine are present (34 in total) and are scattered in four genomic loci as it is usually the case for fluorescent pseudomonads (Ravel and Cornelis 2003). *Pseudomonas baetica* also produces a second siderophore/metallophore, yersiniabactin, which was described in *Yersinia pestis* and in other Enterobacteriaceae (Chaturvedi *et al.*^2012^). Yersiniabactin shares some biosynthesis steps with pyochelin, the second siderophore of *P. aeruginosa* and is able to bind copper with high affinity. It has been also described in some *P. syringae* and *P. avellanae* strains (Jones and Wildermuth 2011; Marcelletti and Scortichini 2015). Interestingly, while most pyoverdine genes have their closest homologs in *P. koreensis*, all, but one, yersiniabactin genes of *P. baetica* show a close identity with the yersiniabactin genes from *P. azotoformans*, which could be indicative of horizontal gene transfer. The genome contains 27 genes coding for a TonB-dependent receptor, including the *fpvA* gene for the ferripyoverdine uptake, and two *fyuA* genes for the uptake of ferri-yersiniabactin. However, two of these genes are truncated and a probable frameshift is evident for one receptor gene. Noteworthy is the presence of six genes annotated as coding for a TonB protein. As a matter of comparison, only three are present in *P. aeruginosa* (Cornelis, Matthijs and van Oeffelen 2009). Another surprising discovery is the absence of *feoABC* genes for the uptake of Fe^2+^ in *P. baetica*. This function is probably secured by the products of the *efeUOAB* operon, first described in *E. coli* O157:H7, which is involved in the uptake of ferrous iron in the periplasm where it gets re-oxidized (Cao *et al.*^2007^).

## DISCUSSION

Whole-genome sequencing has become ubiquitous in microbiology and access to the technology is now widespread. Here, we show that combining two relatively cheap and user-friendly methods (Ion Torrent Semiconductor and Oxford Nanopore MinION sequencing) enables genome assembly of a relatively large bacterial genome to a finishable state. Our phylogenetic analyses show that *P. baetica* is closely related to *P. koreensis* in the *P. fluorescens* group. However, our data show that the *P. fluorescens* group taxonomy is in need of review. Given the close relationship of *P. baetica* a390T to *P. irchel* 3e19, we propose that 3e19 is a strain of *P. baetica.* Although both are aquatic isolates, Irchel 3e19 was isolated from fresh water and *P. baetica* a390T is marine (López *et al.*^2012^; Butaitė *et al.*^2017^). Our data show that the ability to grow in marine salt concentrations is common in the genus *Pseudomonas*. This, combined with the origin of Irchel 3e19 suggests that *P. baetica* a390T is not a marine-restricted organism and is likely to be an opportunist pathogen of marine fish, possibly associated with high fish densities in aquaculture facilities. On the other hand, the presence of a T3SS IN BOTH *P. baetica* A390T and Irchel 3E19 (Prof. Rolf Kummerli, personal communication) suggests that these organisms are not merely benign environmental strains. The *P. baetica* genome shows evidence of genome plasticity and contains a variety of genomic islands and prophages as is the case with other pseudomonads including *P. aeruginosa* (Klockgether *et al.*^2011^). The diverse iron acquisition and membrane transport systems encoded in the *P. baetica* genome are consistent with an organism that is highly adaptable to a range of environments. We find no genomic evidence of known QS systems found in other pseudomonads, but our observations that *P. baetica* is capable of protease secretion and swarming suggest that it may engage in social behaviour.

## SUPPLEMENTARY DATA

Supplementary data are available at *FEMSLE* online.

Supplementary Data

## FUNDING

Work in NPT’s lab has been funded by Biotechnology and Biological Sciences Research Council grant BB/K019600/1 and Chief Scientists Office grant TCS/16/24. AB is supported by a PhD Studentship funded by the Industrial Biotechnology Innovation Centre and the University of Strathclyde. TS was supported by a PhD studentship funded by the Iraqi Government. MG was supported by a postdoctoral fellowship from FWO Vlaanderen (12M4618N). CL was supported by an SB PhD fellowship from FWO Vlaanderen (1S64718N). JB was supported by a grant from Fundação para a Ciência e a Tecnologia (SFRH/BD/104095/2014). NPT would like to personally thank all of the contributors to this paper who have made this novel collaboration an open and pleasurable experience. AM and ED were supported by the Biotechnology and Biological Sciences Research Council-funded South West Biosciences Doctoral Training Partnership (BB/M009122/1).

***Conflict of interest.*** None declared.
