# Supplementary material for: Community-led comparative genomic and phenotypic analysis of the aquaculture pathogen Pseudomonas baetica a390T sequenced by Ion semiconductor and Nanopore technologies
Source: FEMS Microbiol Lett. 2018 Mar 22;365(9):fny069. doi: 10.1093/femsle/fny069 (PMC5909648; doi:10.1093/femsle/fny069)
Supplement: Supplementary Data [file fny069_supp.zip › Final Supplementary figures.docx]

Supplementary figures

**
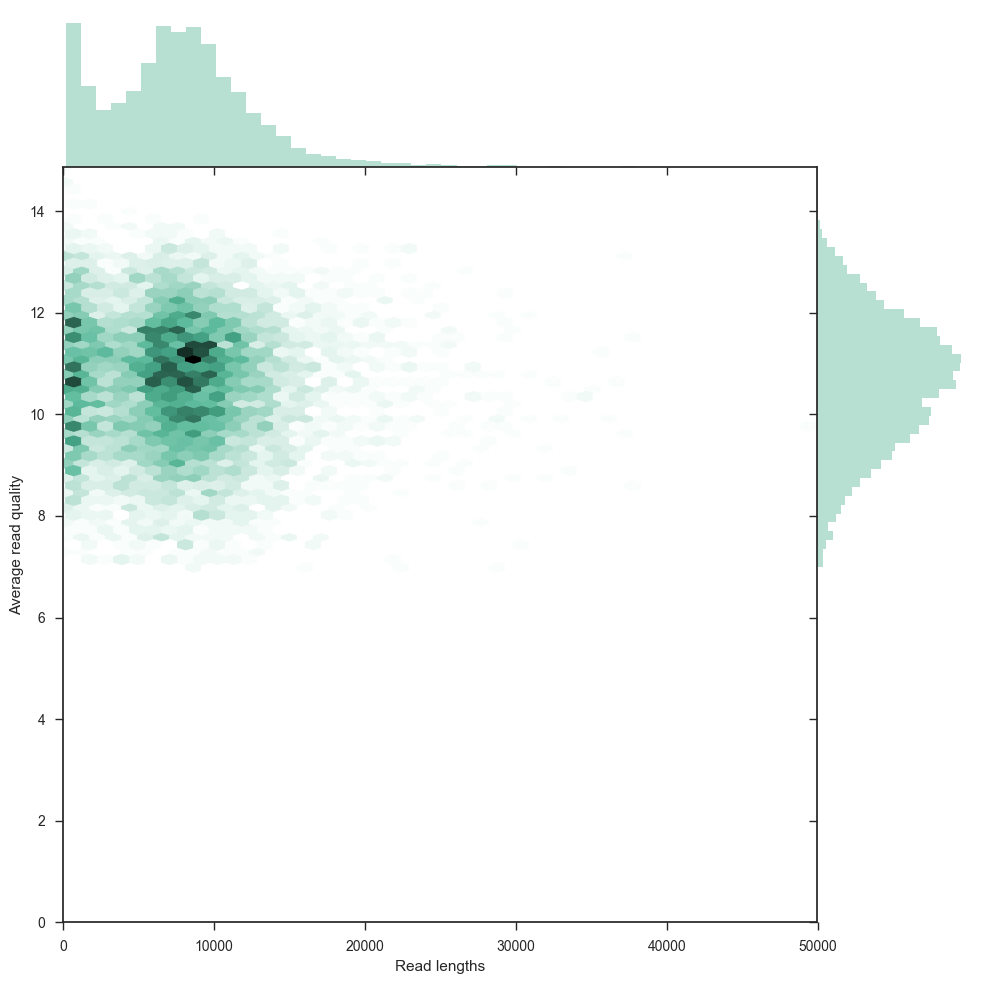
**

Supplementary figure 1: Plot of the quality scores and length distributions for the Nanopore reads.


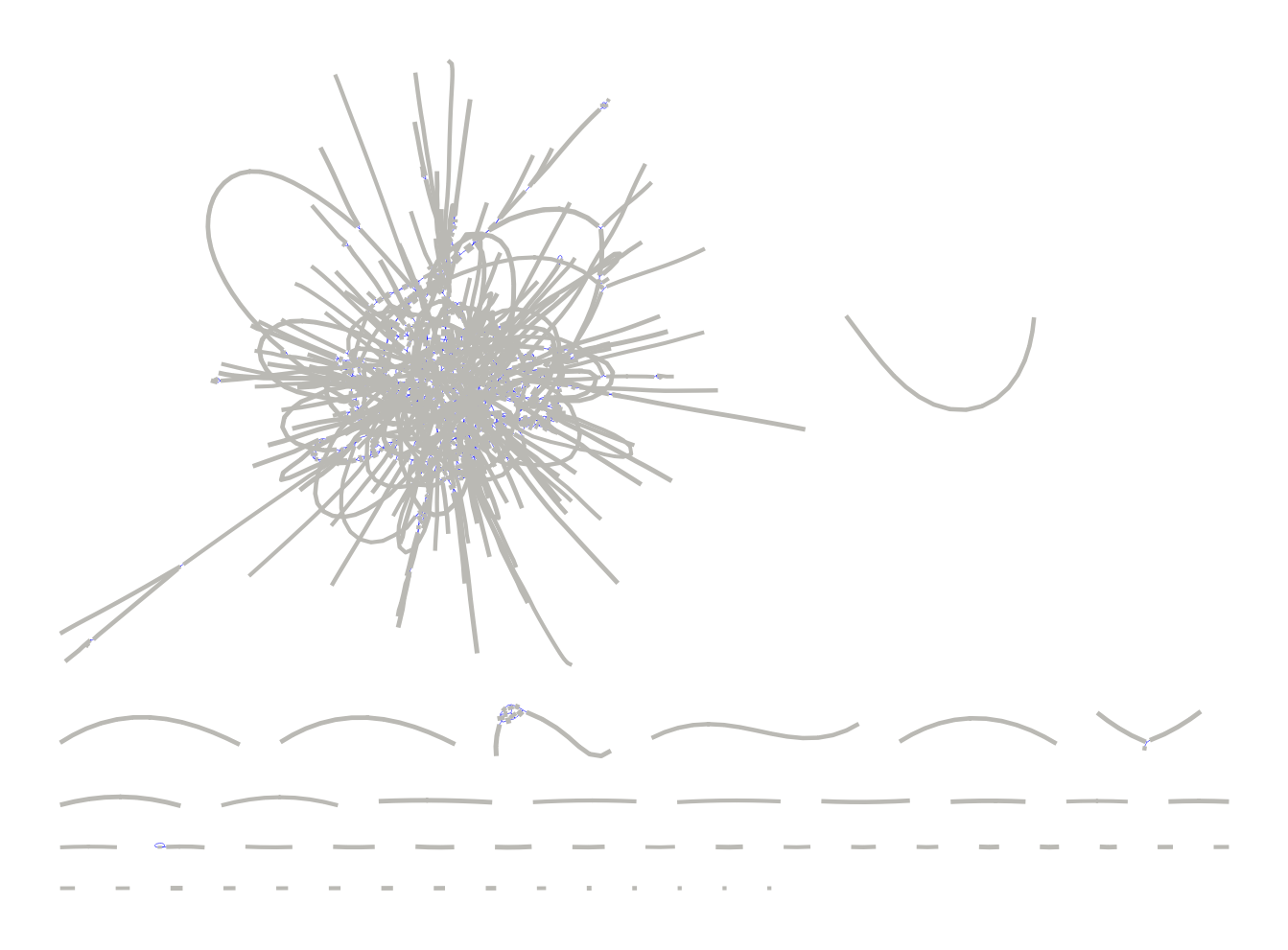


Supplementary figure 2: Bandage visualisation of the Ion Torrent reads assembly graph generated by SPAdes reveals a very fragmented reconstruction of the genome. This is linked to the presence of repetitive regions in the chromosome and/or plasmids that cannot be resolved using short read sequencing.


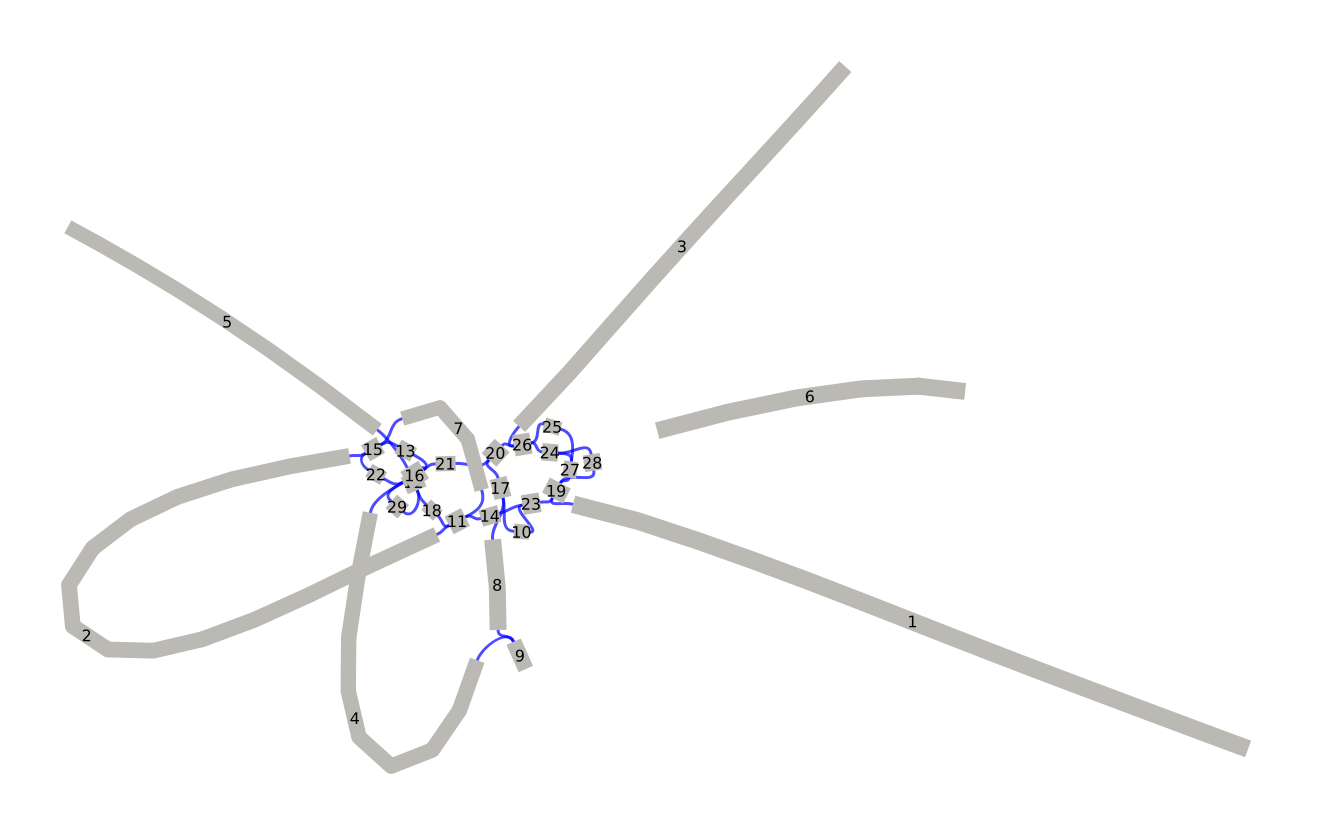


Supplementary figure 3: Bandage visualisation of the hybrid assembly graph generated by Unicycler, where long reads have been used to scaffold the contigs. This assembly show less fragmentation when compared to that of supplementary figure 2. The number of contigs in the assembly is reduced by an order of magnitude (see table 1).

**

Supplementary figure 4: Swimming, swarming and twitching motility of *P. baetica* compared with other members of the genus.

Supplementary figure 5: Digestion of azocasein by *P. aeruginosa, P. baetica, P. fluorescens* and *P. putida* as measured by observance of OD at 440 nm.


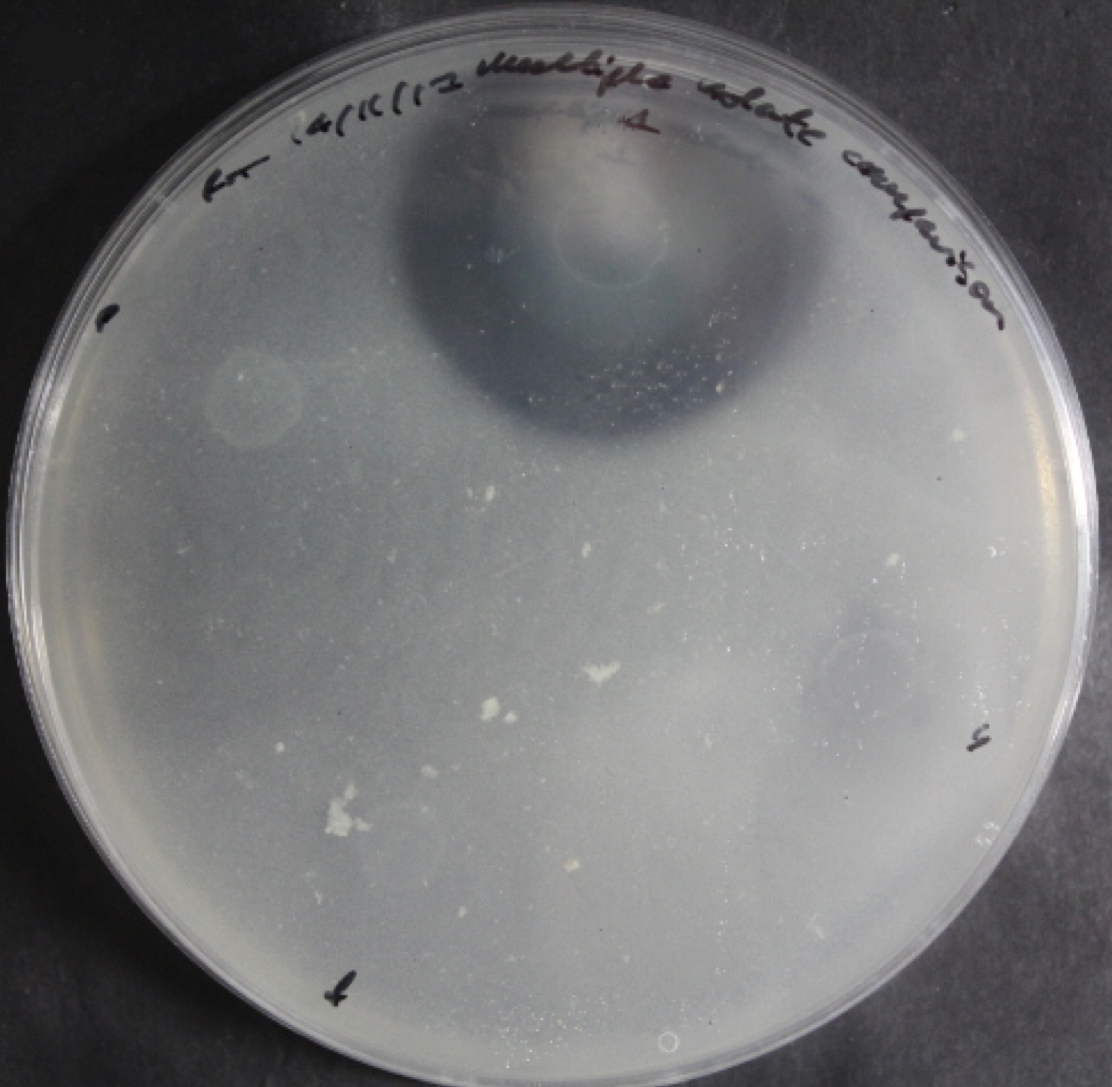

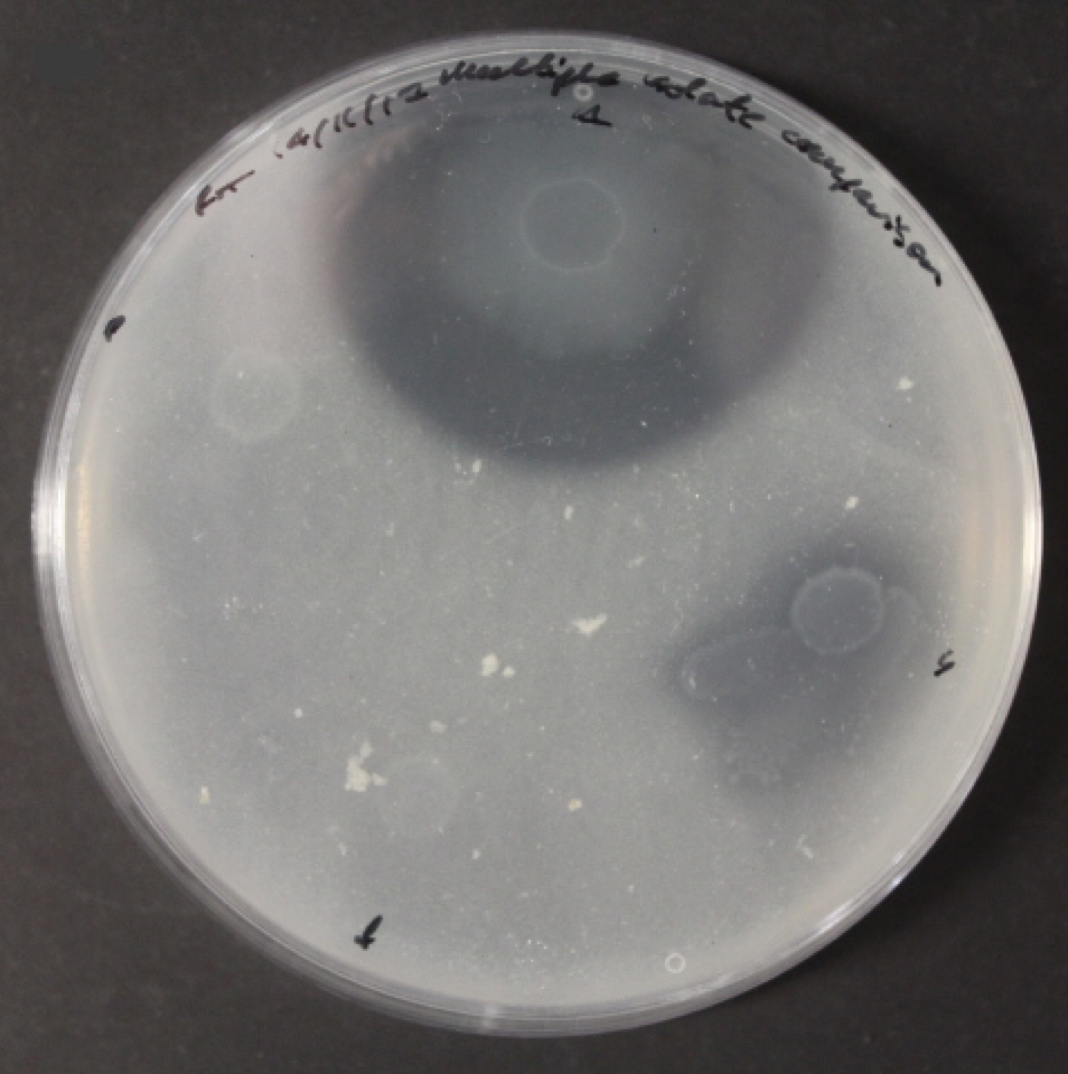


a.

b.

Supplementary figure 6: In each case strains are arranged as follows; Top=*P. aeruginosa* Right=*P. baetica* Bottom=*P. fluorescens* Left=*P. putida* (*a*) Halo formation on MOPS minimal milk agar after 48hrs. (*b*) Halo formation on MOPS minimal milk agar after 72hrs


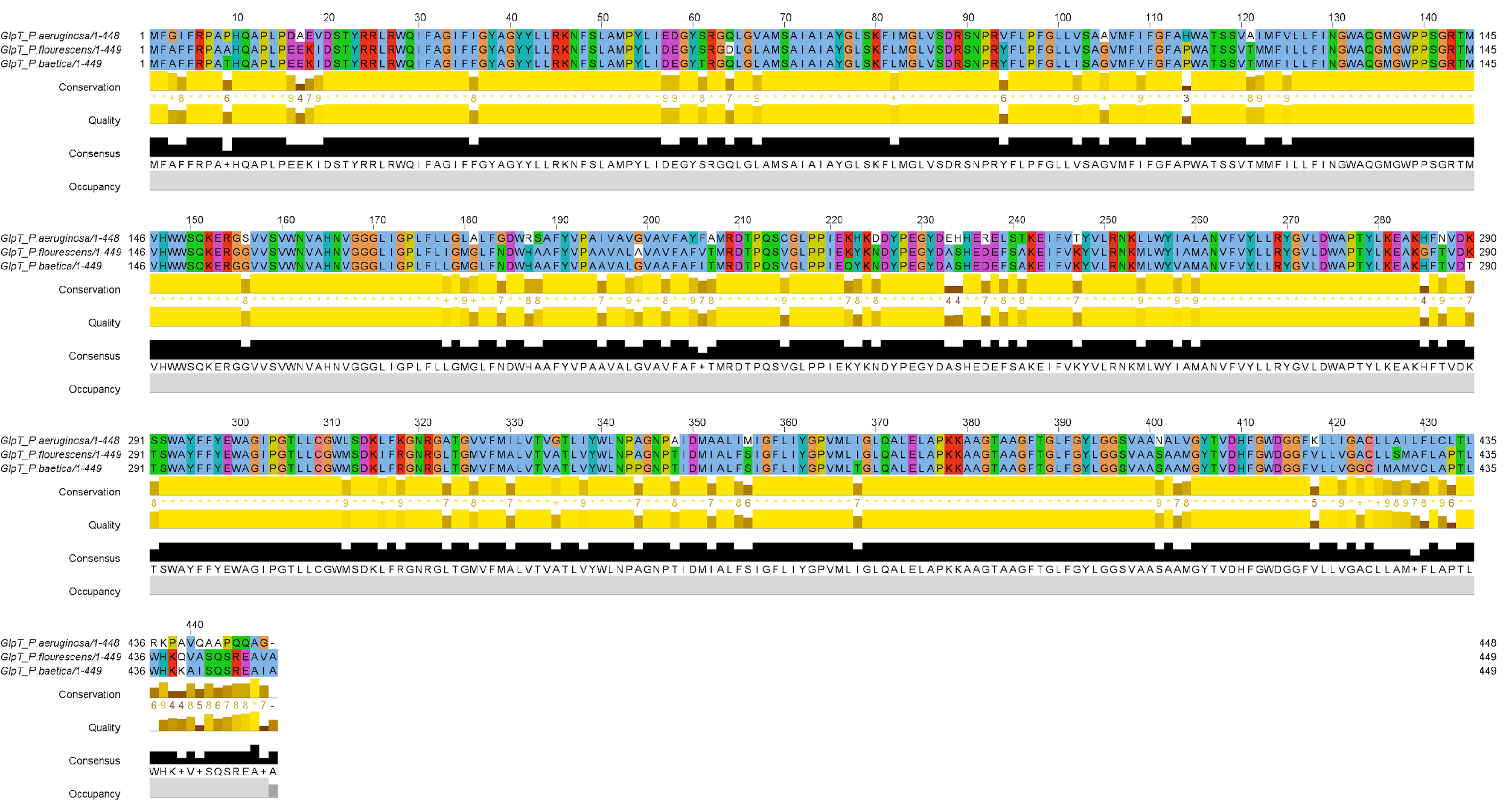


Supplementary figure 7: Comparison of *P. baetica* GlpT amino acid sequence to GlpT from other pseudomonads with confirmed GlpT functionality.

Supplementary figure 8: Growth presented as a percentage of control for *P. aeruginosa, P. baetica, P. fluorescens* and *P. putida* at 24 hours after inoculation.

Supplementary figure 9: Growth presented as a percentage of control for *P. aeruginosa, P. baetica, P. fluorescens* and *P. putida* at 72 hours after inoculation.
