# Supplementary figures and images for: Community-led comparative genomic and phenotypic analysis of the aquaculture pathogen Pseudomonas baetica a390T sequenced by Ion semiconductor and Nanopore technologies
Source: FEMS Microbiol Lett. 2018 Mar 22;365(9):fny069. doi: 10.1093/femsle/fny069 (PMC5909648; doi:10.1093/femsle/fny069)

## Slide 1
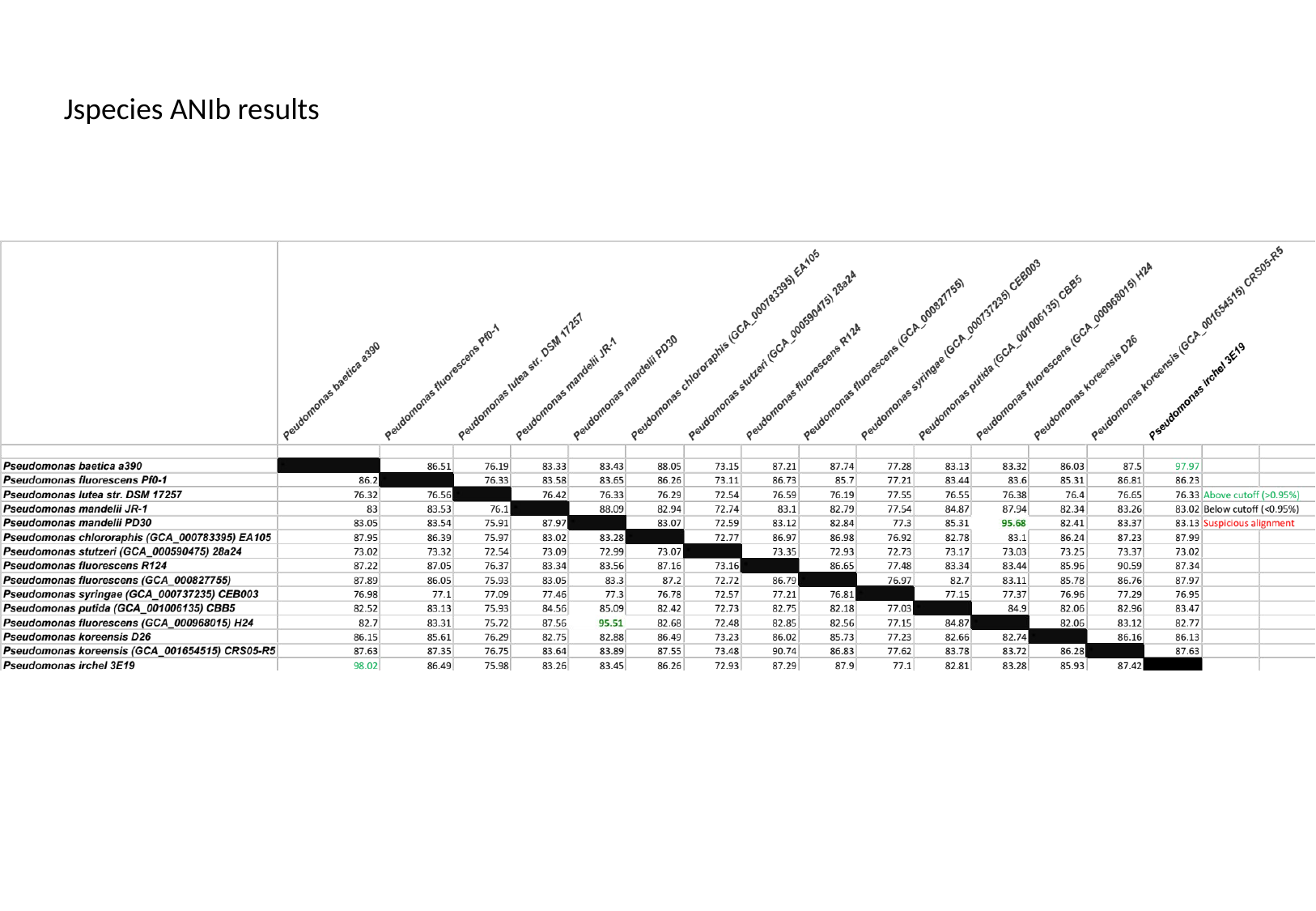

Jspecies ANIb results

## Slide 2
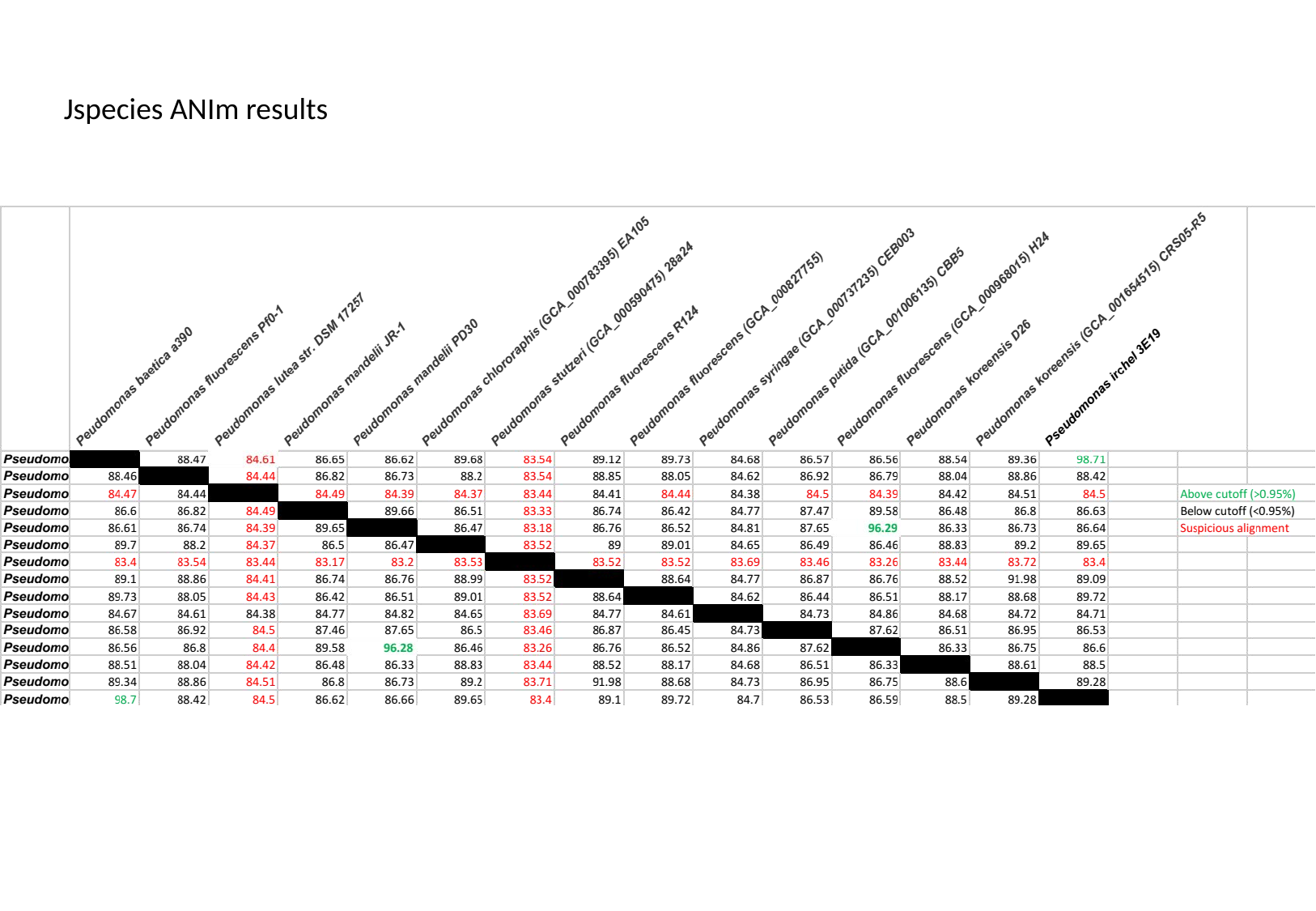

Jspecies ANIm results

## Slide 3
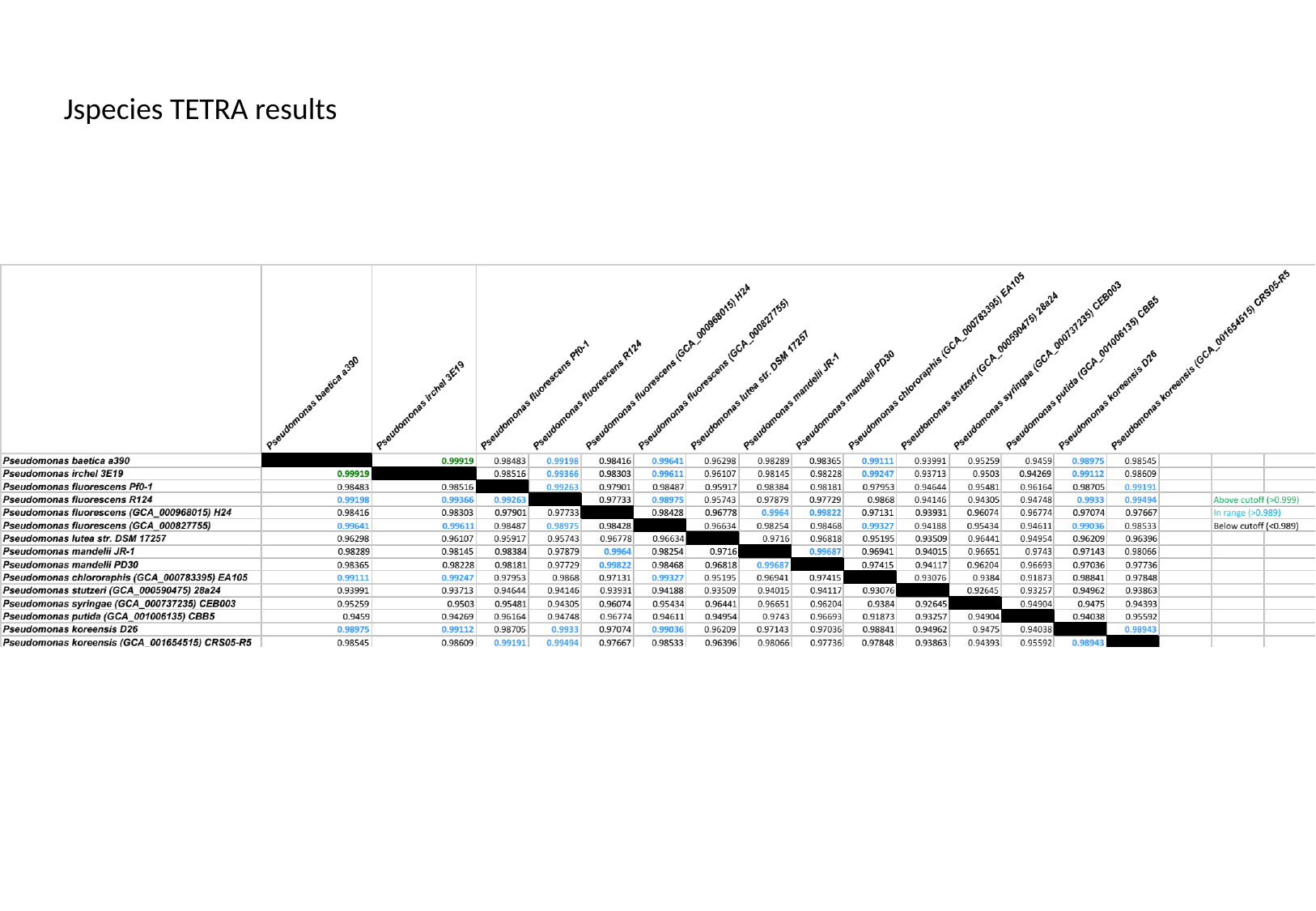

Jspecies TETRA results

Supplement: Supplementary Data [file fny069_supp.zip › Supplementary ANI tables.pptx]
